# Simple, fast and reliable CE method for simultaneous determination of ciprofloxacin and ofloxacin in human urine

**DOI:** 10.1038/s41598-022-11747-y

**Published:** 2022-05-11

**Authors:** Izabella Kośka, Krystian Purgat, Paweł Kubalczyk

**Affiliations:** 1 Department of Environmental Chemistry, Faculty of Chemistry, University of Lodz, Pomorska 163, 90-236 Lodz, Poland; 2 Doctoral School of Exact and Natural Sciences, University of Lodz, Banacha 12/16, 90-237 Lodz, Poland

**Keywords:** Drug regulation, Pharmaceutics, Toxicology, Environmental chemistry, Nutrition, Patient education, Analytical chemistry

## Abstract

A simple, fast, and accurate capillary zone electrophoresis method has been developed for the determination of ciprofloxacin and ofloxacin. This method uses liquid–liquid extraction. Therefore, it is characterized by a very simple procedure of sample preparation but at the same time satisfactory precision and accuracy. The extraction process of the same urine sample was repeated three times. The extraction protocol was performed each time for 15 min with 1 mL of dichloromethane and chloroform mixture in a 3:1 volume ratio. A 0.1 mol/L phosphate-borate buffer (pH 8.40) was selected as the background electrolyte. UV detection was performed at 288 nm. The separation was carried out at a voltage of 16 kV, at a temperature of 25 °C. Experimentally evaluated LOQ values for ciprofloxacin and ofloxacin were 0.2 nmol/mL urine and 0.05 nmol/mL urine, respectively. For both analytes the calibration curves exhibited linearity over the entire tested concentration range of 1–6 nmol/mL urine. The precision of the method did not exceed 15%, and the recovery was in the range of 85–115%. The developed and validated procedure was applied to analyze human urine for the content of ciprofloxacin and ofloxacin.

## Introduction

Nowadays, we observe an increase in consumer interest in the topic of healthy eating. More and more people are aware that diet has an impact on human health. Consumers pay more attention to what they eat and more often read the ingredients listed on the product labels. Unfortunately, food could also contain compounds that are not labeled on a package. These are, for example, animal food additives or medicines given to animals. It is commonly known that antibiotics are increasingly being used in animal husbandry due to the need for rapid growth of animals^1,2^. Excretion of the residues of these compounds with animal urine and feces leads to environmental pollution^1,3,4^. Their use carries a risk to people because they negatively affect human^1–3,5,6^. Long-standing exposure of humans and the environment to antibiotic residues causes the so-called antibiotic resistance, which is one of the most serious global public health problems^1–7^. Soon after the entry of antibiotics to widespread use, resistant and multiresistant strains of the bacteria began to spread rapidly. This is a very unfavorable phenomenon because infections caused by antibiotic-resistant bacteria are very difficult to cure, sometimes even impossible^5^. Therefore, maximum residue limits have been established for veterinary antibiotics in food of animal origin^1,2,6^.

**Figure 1:**
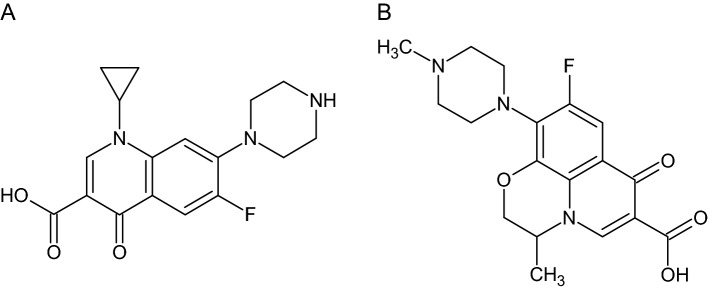
Structures of (**A**) ciprofloxacin, (**B**) ofloxacin.

Fluoroquinolones (FQLs) are a group of compounds that are often used in the treatment of animals. FQLs are synthetic antibiotics derived from diaminopyrimidine^2,3^. They are considered broad-spectrum antibiotics due to their activity against Gram-positive and Gram-negative bacteria, as well as some anaerobes^2,5,6,8,9^. Representatives of FQLs are ciprofloxacin (Cpx) and ofloxacin (Ofx). The Cpx and Ofx structures are shown in Fig. 1

They belong to fluoroquinolone compounds and commonly occur in food of animal origin^3^. Cpx and Ofx are derivatives obtained by the introduction of a fluorine atom at the 6th position of the quinoline ring and at the 7th position of the piperazine ring. These compounds are highly effective in the treatment of several diseases, such as bacterial infections of the gastrointestinal tract and urinary tract^10^. Cpx has 12 approved applications by the US Food and Drug Administration (FDA) in the treatment of humans and animals, but is often used for unapproved applications. Cpx was patented in 1983 by Bayer A.G. for the first time, and then in 1987 Cpx was approved by the FDA^3^. On the other hand, ofloxacin was approved by the FDA in 1992 after being reported by the Johnson Pharmaceutical Research Institute^11^.

Capillary electrophoresis (CE) is a powerful analytical tool characterized by a short analysis time, high separation efficiency, and very low reagents, harmful organic solvents and sample consumption^12^. Despite many advantages, it has limitations, just like other techniques. A commonly known disadvantage of CE is the relatively high limit of quantification (LOQ). It is the result of a very small volume of sample introduced into the capillary and the short optical pathway equal to the inner diameter of the capillary^13,14^.

Nowadays, several analytical methods have been developed for the determination of Cpx and Ofx in various matrices, for example, HPLC methods with UV detection for the determination of FQLs in human plasma^15^, in urine and plasma^16^, and in wastewater^17^. More HPLC methods with mass spectrometry (MS) allow to determine FQLs in animal products^18,19^ or wastewater^20,21^. Several CE methods have also been developed to determine these compounds in animal products^22^ or human urine^5^.

In this protocol for simultaneous CE determination of Cpx and Ofx, liquid–liquid extraction was used as a concentration and purification step during sample preparation.

This method is based in part on our previously developed method for the determination of fluoroquinolones in animal tissues^23^. However, it should be noted that urine is a very complex aqueous matrix that contains significantly varying concentrations of organic compounds, inorganic salts, and trace amounts of biomacromolecules. The presence of these compounds makes it necessary to properly prepare the sample before its analysis. The content of inorganic salts significantly changes the conductivity of the sample and may alter the separation in capillary electrophoresis. Thus, in this work, due to the change in the sample matrix, the entire methodology, i.e., the sample preparation procedure and the CE analysis conditions, was significantly modified. To the best of our knowledge, the literature does not describe the CE method for the determination of ciprofloxacin and ofloxacin in human urine with the use of in capillary preconcentration based on the mechanism of transient pseudo-isotachophoresis.

## Results and discussion

### Optimization of electrophoretic conditions

#### Buffer selection

Four electrolyte solutions, i.e., 0.1 mol/L borate buffer (pH 8.5), 0.1 mol/L sodium tetraborate, 0.1 mol/L phosphate buffer (pH 8.5) and 0.1 mol/L phosphate-borate buffer (pH 8.5) were tested as BGE. The best peaks parameters (height and area, repeatability, and resolution) were obtained using phosphate-borate buffer. Then, the concentration and pH of this buffer were optimized.

#### Optimization of the concentration and pH of BGE

The influence of BGE concentration on the height, area and shape of the peaks, resolution, and repeatability of the sizes of the analytical signals were checked. The concentration of phosphate-borate buffer was checked in the range of 0.05 to 0.2 mol/L. The highest peaks were obtained for 0.1 mol/L phosphate-borate buffer, so this concentration was used for the next experiments. Subsequently, 0.1 mol/L phosphate-borate buffer was tested in the 8.00 to 9.00 pH range with 0.25 increments. As one can see in Fig. S1, the highest signals were obtained for pH 8.25, but unfortunately at this pH the baseline separation of Cpx and Ofx peaks was not achieved. Therefore, to improve the resolution between the peaks, we decided to use a 0.1 mol/L phosphate-borate buffer at pH 8.40.

#### Optimization of capillary temperature

The influence of capillary temperature on peak size was checked at 8 temperature values in the range of 20–27 °C. As expected, migration times increased with increasing temperature, while 25 °C was chosen as the optimal value. At this capillary temperature, the resolution and size of the peaks were also satisfactory.

#### Optimization of separation voltage

The relationship between current and applied voltage was tested to determine maximum voltage to avoid excessive Joul’e heating, which is undesirable effect during electrophoretic analyzes. However, application of higher voltage shortens the migration time of analytes and improves resolution. The maximum value of the separation voltage was set at 23 kV. Thanks to this baseline separation of Cpx and Ofx was achieved. However, in the next stage of the experiment, we decided to add an in-capillary preconcentration step based on the mechanism of transient pseudo-isotachophoresis. The addition of acetonitrile to the sample significantly reduces its conductivity and the voltage of 16 kV appears to be more favorable.

### Optimization of extraction procedure

#### Selection of buffer pH for sample preparation

Proper choice of the pH of the sample is a very important parameter during optimization of the extraction process. The pH value should be carefully selected because the analytes should not have charge when nonpolar organic solvent is used for extraction. In this case, lack of charge facilitates diffusion of molecules into the nonpolar organic solvent and enhances efficiency of extraction. For the selection of an optimal extraction pH, knowledge of the p*K*a value of the analyte is very helpful. Cpx p*K*a values are 8.80 for nitrogen in the piperazinyl ring and 6.00 for the carboxylic acid group, and the Ofx p*K*a values for the ammonium form and for the carboxylic function are 8.28 and 6.10, respectively^22^. Therefore, in this study, the optimization of the pH of the sample was studied by adding 0.2 mol/L phosphate buffer to urine at various pH ranges of 6.50 to 8.00. In Fig. S2 the highest efficiency of the extraction process and satisfactory reproducibility were observed when phosphate buffer was used at pH 7.00.

#### Buffer:urine ratio

The influence of the volume ratio of buffer to urine on extraction efficiency was also checked. The following buffer:urine ratios were checked: 1:2, 1:1 and 2:1. It can be clearly seen in Fig. S3 that the highest signal parameters were obtained when the volume of urine sample was 2 times more than the volume of buffer.

#### Selection of organic solvent

During the choice of organic solvent as the acceptor phase, it is very important that the organic solvent must be immiscible with the aqueous solution and also exhibit good selectivity towards the analyte to obtain the highest efficiency and repeatability of extraction^13^. In this study, the following organic solvents and their mixtures were tested: chloroform, dichloromethane, toluene, hexane, ethyl acetate, chloroform:toluene (1:1, v/v), dichloromethane:toluene (1:1, v/v) and dichloromethane:chloroform (1:1, v/v). In Fig. S4 the best results have been obtained for the mixture of dichloromethane and chloroform. Therefore, in the next step, the effect of various ratios of the mixture on the extraction efficiency was investigated. The following mixtures were checked: dichloromethane, dichloromethane:chloroform (3:1), dichloromethane:chloroform (2:1), dichloromethane:chloroform (1:1), dichloromethane:toluene (2:1), dichloromethane:toluene (1:1). The results showed that the highest extraction efficiency was obtained for the dichloromethane and chloroform mixture in the ratio 3:1 (v/v) (S5). Therefore, this solvent mixture was used as the acceptor phase. Then the volume of the organic phase was selected.

#### Selection of organic solvent volume

The influence of solvent volume on extraction efficiency was tested. For this purpose, the following volumes of dichloromethane and chloroform (3:1) mixture were used for the research: 600, 800, 1000, 1200 and 1400 µL. The highest extraction efficiency was observed when 1000 µL of organic solvent was added (S6). An increase in efficiency of the extraction process was not observed with the addition of larger volumes of the acceptor phase. So finally, it was decided that a volume of 1000 µL would be used as the acceptor phase.

#### Optimization of extraction time

The extraction time needed to achieve the highest efficiency was optimized in the range of 5–30 min. The efficiency of this process did not change significantly and after 15 min it remained at a similar level. With the extraction time of 15 min, the best reproducibility was also obtained. Therefore, 15 min of extraction was chosen.

#### Salt addition

It was checked whether the addition of NaCl can influence the extraction efficiency. NaCl dissolved in water increases the dielectric constant of water, which expands the differences between the hydrophilic and hydrophobic phases, and consequently significantly simplifies phases separation. In the beginning, it was checked whether the addition of 5% NaCl to the sample would increase the extraction efficiency. In this case, changes in the heights of the analytical signal were not observed. So, the higher addition of NaCl, i.e., 15%, was tested in the urine sample. It was observed that the analytical signals were higher; therefore, it was considered that a 15% addition of NaCl was further utilized in the sample preparation procedure. A higher addition of NaCl did not give a positive effect, because during the extraction there were difficulties with phase separation.

#### The number of extractions

The effect of the number of repeated extractions on its efficiency was also checked. For this purpose, 1-, 2-, 3-, and fourfold extraction was performed. It was found that the more times the extraction process is carried out, the higher efficiency of extraction is obtained. However, when the extraction was carried out four times, the efficiency does not change significantly, and the time of sample preparation elongates excessively. Therefore, the three-fold extraction was selected as the optimal.

#### Selection of solvent to dissolve the evaporation residue

To verify the dissolution of the analytes and select which mixture will provide the highest analytical signals after evaporation of the organic extract to dryness, the residue was dissolved in 50 µL of a mixture of methanol and 0.1 mol/L HCl in the following volumetric ratios of methanol and HCl: 1:1, 1:3, 3:1, 1:0, 0:1. Before the selection of the appropriate volumetric ratio of methanol to HCl, the dissolution of Cpx and Ofx in various concentrations of HCl was studied. It was observed that both analytes dissolve very well in 0.1 mol/L HCl. The reason of the addition of methanol is that methanol is characterized by the high viscosity, which simplifies mixing a small volume of the solution in a polypropylene tube. The highest signals were obtained with methanol in a 0.1 mol/L HCl ratio of 1:1 (v/v), and this mixture was used to dissolve the residue after evaporation. Then it was decided to additionally concentrate analytes inside the separation capillary in the CE system. For this purpose, methanol was changed to acetonitrile and 0.1 mol/L HCl was changed to 0.01 mol/L NaOH. The influence of the volume ratio of these compounds on the size of analytical signals was investigated. The following ratios of acetonitrile to NaOH (v/v) were tested: 1:1, 2:1, 3:1, 4:1, 5:1. The best results (the best reproducibility and the highest concentration of analytes) were obtained when the mixture was used in a 3:1 volumetric ratio; further increasing the volume of acetonitrile did not improve the results.

### Influence of the matrix on the slope of the calibration curve

To study the effect of the matrix on the slope of the calibration curves, two urine samples taken from different apparently healthy volunteers were prepared in the following dilutions: 2:1, 1:1, 1:2, 1:3 and 1:7 (urine: 0.2 mol/L phosphate buffer, pH 7.00). The three-point curves were constructed in triplicate (shown in Fig. S7). For each urine dilution, the three-point relationships of the analytical signal and the concentration of the analytes showed similar slope factors. Therefore, it was concluded that the matrix effect has a small impact on the analytical signal and therefore the sample was diluted in the 2:1 ratio in further studies. The repeatability of the slope coefficients of the calibration curves was subsequently tested in urine from a larger number of people. For this purpose, 5-point calibration curves were prepared in 3 series on urine from different people. The slopes of the calibration curves for ciprofloxacin were as follows: 2.8833, 3.5083, 3.1333, 2.5917, 3.025 (CV = 9.94%) and for ofloxacin they were as follows: 24.325, 24.892, 21.275, 28.058 and 25.925 (CV = 9.94%). According to the EMA guide^24^, we find that there is no matrix influence as these ratios do not differ more than 15%.

### Extraction repeatability

Repeatability of extraction was studied by preparing 3 urine samples with different concentrations of Cpx and Ofx. Stock standard solutions of Cpx and Ofx were prepared at concentrations of 3000 nmol/mL in 0.1 mol/L HCl and used for sample spiking with Cpx and Ofx to obtain analytes concentration of 8, 18, 30 nmol/mL urine. Each sample of working solution was prepared in triplicate. After the analysis of the urine extract, the relative standard deviation (RSD) was utilized to estimate the repeatability of the extraction procedure. For both analytes, RSD did not exceed 15%. The detailed data are presented in Table 1.

**Table 1:** Repeatability of ciprofloxacin and ofloxacin extraction.

Concentration* (nmol/mL urine)	RSD (%)
**Ciprofloxacin**	
8	4.6
18	3.0
30	5.5
**Ofloxacin**	
8	5.7
18	6.1
30	2.9

*n = 3.

### Sensitivity enhancement factor

The sensitivity enhancement factor (SEF) can be used to determine the degree of concentration of the analyte with the developed method. It determines the efficiency of extraction by comparing the size of the analytical signal obtained with the method using the concentration of the analyte and the signal without the extraction step. In this work, the following formula was used to calculate the SEF coefficient:

$$SEF = \frac{{h_{after\, extraction} }}{{h_{without\, extraction} }} \cdot \frac{{C_{without\, extraction} }}{{C_{after\,extraction} }}$$

where h_after extraction_—peak height of analyte for urine analysis after extraction, h_without extraction_—peak height of analyte for urine analysis without extraction, C_after extraction_—concentration of analyte in urine sample, which was analyzed with extraction step, C_without extraction_—concentration of analyte in urine sample, which was analyzed without extraction step.

Urine samples were prepared according to the procedure described in section “Calibration of the method”, each sample was prepared in triplicate, and the SEF’s for Cpx and Ofx calculated from the above equation were 142.4 and 216, respectively.

### Calibration and other validation data

The method developed for the simultaneous determination of Ofx and Cpx in human urine was validated under optimized conditions according to the criteria for biological sample analysis^11^. The limit of detection (LOD) was experimentally determined as the concentration that corresponds to the analytical signal 3 times higher than the baseline noise, while the limit of quantification (LOQ) was the concentration that corresponds to the signal 9 times higher than the baseline noise^25^. The LOD and LOQ values evaluated for Cpx were: 0.05 and 0.2 nmol/mL urine and for Ofx were 0.01 nmol/mL urine and 0.05 nmol/mL urine, respectively. The LOD and LOQ of the developed method are lower than for CE procedure^26^ and CIEF method^27^ but higher than for UPLC-MS/MS method^1^ and MSPE-HPLC–MS procedure^28^ (Table 2).

**Table 2:** Comparison the LOD and LOQ values of the described method with published methods.

Method	LOD		LOQ	
	Cpx	Ofx	Cpx	Ofx
CE^26^	12.83 nmol/mL* (0.00425 mg/mL)	No data	37.73 nmol/mL* (0.0125 mg/mL)	No data
CIEF^27^	1.03 nmol/mL* (0.34 µg/mL)	3.32 nmol/mL* (1.20 µg/mL)	No data	No data
UPLC-MS/MS^1^	1.38 × 10^–5^ nmol/mL* (0.005 ng/mL)	1.38 × 10^–5^ nmol/mL* (0.005 ng/mL)	4.14 × 10^–5^ nmol/mL* (0.015 ng/mL)	4.14 × 10^–5^ nmol/mL* (0.015 ng/mL)
MSPE-HPLC-MS^28^	3.05 × 10^–5^ nmol/mL* (10.1 ng/L)	8.58 × 10^–6^ nmol/mL* (3.1 ng/L)	1.02 × 10^–4^ nmol/mL* (33.8 ng/L)	2.88 × 10^–5^ nmol/mL* (10.4 ng/L)
Presented method	0.05 nmol/mL	0.01 nmol/mL	0.2 nmol/mL	0.05 nmol/mL

*LOD and LOQ values converted and reported in nmol/mL urine.

Six-point calibration curves were constructed for both Cpx and Ofx in human urine in the range of 1–6 nmol/mL, in triplicate. The calibration curves obtained (Fig. S8) exhibit a linear character in the tested concentration range. The square of the linear correlation coefficient (R^2^) for Cpx was 0.9936 and for Ofx was 0.9987. The equation of the calibration curve for Cpx was y = (7.093 ± 0.285)x − (4.187 ± 0.453) and for Ofx was y = (45.110 ± 0.828)x − (0.511 ± 0.298). The RSD of the points of the calibration curves in urine for Cpx and Ofx ranged from 3.7 to 9.3%, while recovery ranged from 97.5 to 104.5%. These values are in good agreement with the criteria approved for the analysis of biological samples^11^. Then, intra-day and inter-day validation was performed. For this purpose, three concentrations of the quantified compounds within the range of the calibration curve were studied. The first concentration represents the beginning of the calibration curve range, the next concentration was in the middle of the calibration curve range, and the third concentration was near the upper boundary of the calibration curve. Due to satisfactory precision (less than 15%) and recovery (in the range 85–115%), we strongly believe that the developed method can be used to determine Cpx and Ofx in human urine. All validation data are presented in Table 3.

**Table 3:** Validation data—results.

Added* (nmol/mL urine)	Intra-day			Inter-day		
	Found ± SD (nmol/mL urine)	RSD (%)	Accuracy (%)	Found ± SD (nmol/mL urine)	RSD (%)	Accuracy (%)
**Ciprofloxacin**						
1.50	1.42 ± 0.02	1.4	94.8	1.46 ± 0.11	7.8	97.0
2.50	2.25 ± 0.05	2.2	89.9	2.32 ± 0.12	5.3	92.6
4.50	3.89 ± 0.18	4.7	86.4	4.11 ± 0.11	2.6	91.4
**Ofloxacin**						
1.50	1.51 ± 0.12	7.8	100.7	1.53 ± 0.09	6.0	102.1
2.50	2.56 ± 0.18	7.0	102.6	2.49 ± 0.06	2.4	99.6
4.50	4.44 ± 0.12	2.6	98.7	4.47 ± 0.07	1.9	99.4

*n = 3.

The procedure for preparing the urine sample is simple, but quite time consuming, the overall time needed to analyze the urine sample for the Cpx and Ofx content with the use of the above-mentioned procedure equals 68 min. However, during this time, at least 10 samples can be prepared simultaneously (this number corresponds to the number of places on the shaker). The overall analysis time of our methodology is similar to that of the MSPE-HPLC–MS method^28^ (72 min) or SDME-CE procedure^5^ (61 min), but much shorter than in EEM-ANWE approach^10^ (155 min).

### Determination of Cpx and Ofx in human urine

**Figure 2:**
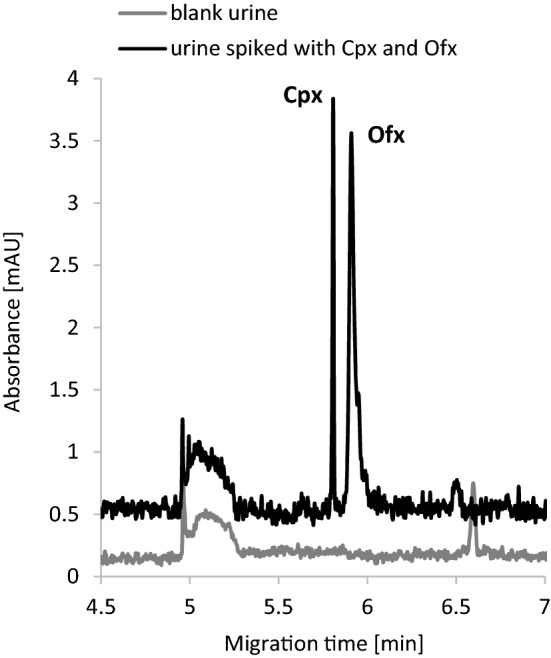
Representative electropherograms obtained for urine and urine spiked with Cpx and Ofx (final concentration 6 nmol/mL urine).

After conducting the whole optimization, calibration, and validation protocol, the analytical procedure was applied to analyze human urine samples for the Cpx and Ofx content. Urine samples were taken from 6 apparently healthy volunteers, spiked with a known amount of Cpx and Ofx to obtain a concentration of 6 nmol/mL urine for both analytes, and prepared according to the procedure described in section “Urine sample preparation”. The samples were then subjected to analysis with the use of capillary zone electrophoresis with concentration in the capillary. All the data are shown in Table 4. Representative electropherograms obtained for urine and spiked urine are shown in Fig. 2. The RSD value exceeds 15% for one urine sample, it may be due to its own properties. The high value of RSD in the case of analysis of one urine sample may result from mistakes made during the sample preparation, mainly incomplete collection of the organic phase during the extraction. In addition, the sample to be analyzed contains acetonitrile, so it could evaporate while it was in the CE apparatus.

**Table 4:** Determination of Cpx and Ofx in human urine—results.

Sample number	Added* (nmol/mL urine)	Found ± SD (nmol/mL urine)	RSD (%)
**Ciprofloxacin**			
1	6.00	6.68 ± 0.57	8.50
2	6.00	5.88 ± 0.57	9.7
3	6.00	6.28 ± 0.57	9.1
4	6.00	6.08 ± 0.29	4.7
5	6.00	5.96 ± 1.03	17.3
6	6.00	7.09 ± 0.57	8.1
**Ofloxacin**			
1	6.00	6.16 ± 0.07	1.10
2	6.00	6.26 ± 0.48	7.6
3	6.00	6.69 ± 0.55	8.0
4	6.00	6.16 ± 0.48	7.8
5	6.00	6.55 ± 0.75	11.5
6	6.00	6.31 ± 0.55	8.7

*n = 3.

### Stability tests

#### Thermal stability

Both Cpx and Ofx concentrations are constant over the whole range of evaporation temperature from 60 to 100 °C (Fig. S8). The Cpx concentration ranges from 11.09 ± 0.35 nmol/mL urine to 12.66 ± 0.27 nmol/mL urine and the Ofx concentration is within the range from 10.09 ± 0.40 nmol/mL urine to 11.03 ± 1.00 nmol/mL urine. Therefore, it can be assumed that if the meat containing FQLs is cooked at a temperature below 100 °C, the tested FQLs are still present in the food. Since Cpx and Ofx are stable at high temperatures and considering that the evaporation time is equal to 15 min, the optimal evaporation temperature was found to be 90 °C.

#### Long-term stability

Experiments on the long-term stability of Cpx and Ofx have shown that these compounds do not degrade over time. As can be seen in Fig. S10, the tested FQLs are stable at room temperature at both light and dark and low temperatures, and their concentration is not affected by thaw. The average Cpx and Ofx content found in urine was 10.44 ± 1.81 nmol/mL urine and 10.12 ± 0.75 nmol/mL urine for samples stored in light, 10.58 ± 1.66 nmol/mL urine and 10.08 ± 1.25 nmol/mL urine for samples stored in the dark, 10.28 ± 1.77 nmol/mL urine and 10.03 ± 0.89 nmol/mL urine during the storage of samples at 4 °C. When samples were stored at − 24 °C and after several freeze–thaw cycles, mean Cpx and Ofx concentrations were 9.35 ± 2.38 nmol/mL urine and 8.85 ± 1.72 nmol/mL urine, respectively.

## Materials and methods

### Instruments

For all experiments, Agilent 7100 CE System (Waldbronn, Germany) coupled with UV–Vis absorbance diode array detector and equipped with automatic injector was used. For separation, bare fused silica capillary (Polymicro Technologies, Phoenix, USA) of total length of 64.5 cm (effective length of 56 cm) and inner diameter of 75 µm was utilized. To measure peak areas, migration times, and other data, Agilent ChemStation software was used. Peaks of these compounds were identified by comparing their spectra in the standard sample with those in the biological sample, as well as by comparing the migration times of analytes on electropherograms of the standard and the real sample. To shake the samples, a vortex was used, in turn, a thermostat was used to evaporate the organic solvent, and the pH of the solutions was adjusted using a pH-meter from Mettler Toledo (Switzerland). The deionized water used for all experiments was purified using a Millipore Milli‐Q‐RG System (Waterford, Ireland).

### Chemicals

Sodium hydroxide (NaOH) and sodium chloride (NaCl) were purchased from POCH (Gliwice, Poland), sodium tetraborate decahydrate (B_4_Na_2_O_7_·10 H_2_O) was obtained from Sigma (Steinheim, Germany), boric acid (H_3_BO_3_), dichloromethane (CH_2_Cl_2_) and methanol (CH_3_OH) were from J.T. Baker (Deventer, The Netherlands). Chloroform (CHCl_3_), toluene (C_7_H_8_), and ethyl acetate (C_4_H_8_O_2_) were purchased from Chempur (Piekary Slaskie, Poland). The hexane was obtained from Lab-Scan (Dublin, Ireland). Standards of analytes, i.e., Cpx (C_17_H_18_FN_3_O_3_) and Ofx (C_18_H_20_FN_3_O_4_) were obtained from Sigma-Aldrich (Saint Louis, Missouri, United States of America). The buffer pH was adjusted by potentiometric titration and degassed using an ultrasonic bath.

### Capillary preconditioning

In case of new capillary, the preconditioning procedure was performed by flushing with 1 mol/L NaOH solution for 20 min, next with 0.1 mol/L solution of NaOH for 20 min, then 2 min with deionized water, and finally with BGE for 30 min. Every next day of work, the capillary was flushed with 1 mol/L NaOH solution for 5 min and 0.1 mol/L NaOH solution for 20 min, then with water for 2 min, and 30 min with BGE. At the end of each day, the capillary was flushed with water for 20 min and the capillary ends were left in the water overnight.

### Electrophoretic conditions

During electrophoretic analysis, 0.1 mol/L (pH 8.40) phosphate-borate buffer was used as BGE. Separation was achieved with a voltage of 16 kV at 25 °C. Hydrodynamic injection of the sample solution was performed at 60 mbar for 30 s. UV–Vis detection was performed at an analytical wavelength of 288 nm for both FQLs.

### Human urine collection

All experiments were carried out according to the Institutional Ethics Approval of the relevant University Committee (Resolution no. 8/KBBN-UŁ/I/2020–2021). To develop the method, human urine samples were collected from apparently healthy volunteers. These samples were centrifuged for 5 min at 12,000 rpm, then the supernatant solution was collected in a polypropylene tube and stored at reduced temperature (− 20 °C).

### Urine sample preparation

To prepare the sample for the extraction procedure, 268 µL of human urine with 0.06 g NaCl was transferred into polypropylene vial and 132 µL of 0.2 mol/L (pH 7.00) phosphate buffer was added (the urine–buffer ratio was 2:1) and the mixture obtained was mixed. Then 1 mL of the dichloromethane and chloroform mixture (3:1) was added and vigorously shaken for 15 min at 3500 rpm. The sample was then centrifuged for 5 min at 12,000 rpm. 1 mL of the organic solvent (lower phase) was collected and evaporated to dryness. The same sample was extracted three times. The residue was dissolved in 50 µL of the mixture of acetonitrile and 0.01 mol/L NaOH (3:1, v/v), introduced to the CE system and analyzed.

### Calibration of the method

Calibration standards for the determination of Cpx and Ofx in urine were prepared by diluting 3000 nmol/mL of Cpx and Ofx with 0.1 mol/L HCl as needed. To calibrate the method, 3 series of solutions with increasing concentrations were prepared. All working solutions were prepared according to the following procedure: 268 µL of human urine with 0.06 g NaCl was added to a polypropylene tube and 132 µL of 0.2 mol/L phosphate buffer (pH 7.00) was added and mixed. Then, the solutions obtained were spiked with the growing amounts of working standard solutions of both Cpx and Ofx to obtain the following concentrations: 1 nmol/mL urine, 2 nmol/mL urine, 3 nmol/mL urine, 4 nmol/mL urine, and 5 nmol/mL urine, 6 nmol/mL urine. The volume of spiking solutions added to all calibration samples was the same. Subsequently, 1 mL of the dichloromethane and chloroform mixture (3:1) was added, the mixture was vigorously shaken for 15 min at 3500 rpm and centrifuged for 5 min at 12,000 rpm. The organic phase was collected (extraction of the same sample was carried out three times), evaporated to dryness, and the residue was dissolved in 50 µL of acetonitrile and 0.01 mol/L NaOH (3:1, v/v), then the sample was introduced to the CE system and analyzed. The peak areas of Cpx and Ofx were plotted versus their corresponding concentrations, and the curves were fitted using a least-squares linear regression analysis.

### Stability tests

For thermal stability experiments, 5 series of urine samples without FQLs content were prepared (according to the procedure described in section “Urine sample preparation”) and spiked with known amounts of working standard solutions of Cpx and Ofx, to provide final concentrations of these compounds equal 10 nmol/mL urine. Each such urine sample was prepared in triplicate. The first series was tested for thermal stability, the second series was subjected to long-term stability tests at room temperature in daylight, another series at room temperature in the dark, and the last two series at reduced temperatures of 4 °C and − 24 °C, respectively. For studies of the thermal stability of Cpx and Ofx samples were evaporated at different temperatures: 60, 70, 80, 90, and 100 °C. However, to test long-term stability, the enriched samples were stored at the above mentioned temperatures and analyzed after 1, 2, 3, 7, and 36 days.

### Ethics declarations

The study was conducted according to the guidelines of the Declaration of Helsinki, and approved by the Ethics Committee of the University of Lodz (protocol code 8/KBBN-UŁ/I/2020–2021).

### Informed consent

Informed consent was obtained from all subjects involved in the study.

## Conclusions

A new, fast and simple analytical procedure for the determination of Cpx and Ofx in human urine with the use of CZE with UV–Vis detection and analytes concentration by in-capillary pseudo-transient isotachophoresis and liquid–liquid sample extraction was developed. This method is characterized by a very simple and a relatively fast preparation of the urine sample. In this study, the mixture of dichloromethane and chloroform (3:1, v/v) was chosen for sample extraction as the organic phase. The extraction process of the same urine sample was carried out three times, each time for 15 min, with 1 mL of the organic solvent. The described procedure is very simple and does not require sophisticated equipment while maintaining relatively high sensitivity. Besides, the developed method is sensitive and precise, and it is characterized by good linearity and accuracy. Considering all these advantages of our method, we strongly believe that it can be implemented in the routine analysis of urine for Cpx and Ofx content.

## Supplementary Information

Supplementary Figures.

## Data Availability

CE data are available from the authors.
