# Supplementary material for: Simple, fast and reliable CE method for simultaneous determination of ciprofloxacin and ofloxacin in human urine
Source: Sci Rep. 2022 May 11;12:7729. doi: 10.1038/s41598-022-11747-y (PMC9095712; doi:10.1038/s41598-022-11747-y)
Supplement: Supplementary file 1 — Supplementary Figures. [file 41598_2022_11747_MOESM1_ESM.docx]

S1. The relationship between the peak height and the pH oh the background electrolyte

S2. The relationship between the peak area and the buffer pH

S3. The effect of the buffer:urine ratio on peak area

S4. The influence of an organic solvent on the peak area

S5. The influence of ratio of dichloromethane and chloroform on the peak area

S6. The influence of the organic layer volume on the peak area

S7. The relationship between the peak area and the concentration of Cpx and Ofx for two different urines in the ratio with the buffer, respectively: A- 2:1, B- 1:1, C- 1:2, D- 1:3, E- 1:7

S8. Calibration curves

S9. Thermal stability of ciprofloxacin and ofloxacin

S10. Stability of ciprofloxacin and ofloxacin on the light, in the dark, at 4 and -24 °C.
